# Enhanced corticomuscular coherence by external stochastic noise

**DOI:** 10.3389/fnhum.2014.00325

**Published:** 2014-05-20

**Authors:** Carlos Trenado, Ignacio Mendez-Balbuena, Elias Manjarrez, Frank Huethe, Jürgen Schulte-Mönting, Bernd Feige, Marie-Claude Hepp-Reymond, Rumyana Kristeva

**Affiliations:** ^1^Department of Neurology, University of FreiburgFreiburg, Germany; ^2^Facultad de Psicologia, Benemérita Universidad Autonoma de PueblaPuebla, Mexico; ^3^Instituto de Fisiologia, Benemérita Universidad Autonoma de PueblaPuebla, Mexico; ^4^Institute for Medical Biometry and Medical Informatics, University of FreiburgFreiburg, Germany; ^5^Department of Psychiatry, University of FreiburgFreiburg, Germany; ^6^Institute of Neuroinformatics, University of Zürich, ETH ZürichZurich, Switzerland

**Keywords:** noise, corticomuscular coherence, stochastic resonance, finger, motor, force, humans

## Abstract

Noise can have beneficial effects as shown by the stochastic resonance (SR) phenomenon which is characterized by performance improvement when an optimal noise is added. Modern attempts to improve human performance utilize this phenomenon. The purpose of the present study was to investigate whether performance improvement by addition of optimum noise (ON) is related to increased cortical motor spectral power (SP) and increased corticomuscular coherence. Eight subjects performed a visuomotor task requiring to compensate with the right index finger a static force (SF) generated by a manipulandum on which Gaussian noise was applied. The finger position was displayed on-line on a monitor as a small white dot which the subjects had to maintain in the center of a green bigger circle. Electroencephalogram from the contralateral motor area, electromyogram from active muscles and finger position were recorded. The performance was measured by the mean absolute deviation (MAD) of the white dot from the zero position. ON compared to the zero noise condition induced an improvement in motor accuracy together with an enhancement of cortical motor SP and corticomuscular coherence in beta-range. These data suggest that the improved sensorimotor performance via SR is consistent with an increase in the cortical motor SP and in the corticomuscular coherence.

## Introduction

Stochastic resonance (SR) is a phenomenon in non-linear systems in which an intermediate level of Gaussian noise enhances the response to weak signals (cf. reviews by Wiesenfeld and Moss, 1995; Gammaitoni et al., 1998; Moss et al., 2004; Ermentrout et al., 2008; Faisal et al., 2008; McDonnell and Abbott, 2009; Aihara et al., 2010; McDonnell and Ward, 2011). The signature of the SR phenomenon is an inverted U-curve with best performance for the intermediate level of noise called optimum noise (ON). In the nervous system SR has been studied in sensory, motor, and sensorimotor systems. The first demonstration of this phenomenon in sensory systems was by Douglas et al. (1993) in the crayfish mechanoreceptors. The first demonstration of SR in the motor system was given by Martinez et al. (2007) at the spinal level. The SR phenomenon has also received considerable attention in sensorimotor control, in particular in balance (Priplata et al., 2002, 2006; Collins et al., 2003; Harry et al., 2005; Costa et al., 2007; Galica et al., 2009; Magalhaes and Kohn, 2011; Mulavara et al., 2011) and force control (Mendez-Balbuena et al., 2012; Trenado et al., 2014). Recent studies have also shown the application of SR in improving the finger's tactile sensitivity in healthy subjects and stroke patients (Enders et al., 2013; Kurita et al., 2013).

The phenomenon of enhanced coherence produced by optimal noise has been also described in theoretical models of neurons (Casado, 1997; Neiman et al., 1997; Pikovsky and Kurths, 1997; Lee et al., 1998; Wang et al., 2000; Zhong and Xin, 2000; McMillen and Kopell, 2003; Kreuz et al., 2006; Torcini et al., 2007; Li and Gao, 2008; Guo and Li, 2009; Jiang and Ma, 2009; Franović et al., 2012; Gao and Wang, 2012; Men et al., 2012) and experimentally in physical systems (Lih et al., 1998; Postnov et al., 1998; Giacomelli et al., 2000; Zhong and Xin, 2000, 2001a,b; Calvo et al., 2001; Zhong et al., 2001; Kortlüke et al., 2002; Li and Li, 2003, 2004a,b,c, 2006a,b; Bahar and Moss, 2004; Wang et al., 2010; Xin and Hou, 2006; Perc, 2007; Ermentrout et al., 2008; Li et al., 2008; Perc et al., 2008; Shi and Lang, 2009; Shi and Luo, 2010). It has been experimentally shown that the SR is related to increased local (within one area) and long-range (between different areas) synchrony. In 2002 we demonstrated for the first time that the central nervous system exhibits internal SR (Manjarrez et al., 2002b). We found in anesthetized cats that the coherence between spinal and cortical somatosensory neurons can be enhanced by the application of a particular level of tactile noise, thus suggesting that spinal and cortical neurons are activated with more synchrony during the noisy tactile sensation. Increased synchrony with SR had also been described by other authors (Kitajo et al., 2003, 2004, 2007; Ward et al., 2006).

But what are the neural mechanisms of this phenomenon in the sensorimotor system? Psychophysical studies (Priplata et al., 2002, 2006; Harry et al., 2005; Costa et al., 2007; Galica et al., 2009; Magalhaes and Kohn, 2011; Mulavara et al., 2011) provided evidence that mechanical noise applied to the feet via vibrating insoles and stochastic electrical stimulation applied of the vestibular organs the vestibular system improved balance in standing position. We recently reported the improvement of precision in force control when an optimum Gaussian noise was added to the manipulandum (Mendez-Balbuena et al., 2012; Trenado et al., 2014). In these two psychophysical experiments the motor task mainly involved the metacarpophalangeal joint of the right index finger, requiring the pseudo-isometric compensation of a low static force (SF). In Trenado et al. (2014) we even demonstrated that broad-band 0–300 Hz Gaussian noise is most effective in improving sensorimotor performance and is most pleasant. We suggested the existence of stronger interaction between internal SR and the externally applied noise when the noise frequency bandwidth includes the frequencies for which the Pacinian receptors are most sensitive. This suggestion was based on Aihara et al. (2010) who had hypothesized that the optimization of the performance by externally applied noise is related to the interaction between externally applied noise and the internal noise of the system. In fact, in the nervous system internal noise exists from the molecular to the behavioral level (Faisal et al., 2008).

The visuomotor task used in our two studies has the advantage that the motor task is less complex than balance and gait, and is well understood in terms of its oscillatory motor activity (Kristeva-Feige et al., 1993; Andrykiewicz et al., 2007; Kristeva et al., 2007; Omlor et al., 2007; Mendez-Balbuena et al., 2012). Several other groups also showed that SF compensation is accompanied by beta-range corticospinal synchronization measured by beta-range corticomuscular coherence (CMC) (Murthy and Fetz, 1992; Sanes and Donoghue, 1993; Conway et al., 1995; Murthy and Fetz, 1996a,b; Baker et al., 1997; Salenius et al., 1997; Halliday et al., 1998; Brown, 2000; Gross et al., 2000; Baker and Baker, 2003; Baker et al., 2006; Perez et al., 2006; Riddle and Baker, 2006; Tecchio et al., 2006; Cheyne et al., 2008; Bressler, 2009; Engel and Fries, 2010; Houweling et al., 2010; Witham et al., 2010).

Therefore, combining our psychophysical SR paradigm with electroencephalographic (EEG) and electromyographic (EMG) recordings should give insight in understanding the neuronal basis of the SR in the sensorimotor system. To this aim we designed an experiment in which we compared motor performance, cortical spectral power (SP) over the motor cortex and CMC in an experimental condition with a broad-band 0–300 Hz Gaussian noise added to the finger manipulandum with another without noise.

Based on our earlier finding about a highest signal-to noise ratio in the somatosensory evoked potentials with ON elicited by a mechanical tactile stimulus we predicted a stronger cortical motor synchrony, reflected in higher cortical motor SP with ON than without (Manjarrez et al., 2002a, 2003). This prediction was also based on the study of Srebro and Malladi (1999) who found a highest signal-to noise ratio in visual evoked potentials for an ON level. Studies on deafferentation which demonstrated that cutaneous input enhanced oscillatory synchrony in the motor system also give support to this prediction (Fisher et al., 2002; Patino et al., 2008).

Synchronous discharge of neurons in the primary motor cortex (Allum et al., 1982; Hatsopoulos et al., 1998) may be more effective in driving spinal motoneurons. Subsequently, Baker et al. (1999) showed that the high beta-range cortical SP was related to a strong corticospinal drive, i.e., to higher CMC. Further, we and others found that higher beta-SP and CMC correlated with better motor performance (Kristeva et al., 2007; Pogosyan et al., 2009; Mendez-Balbuena et al., 2012). Therefore, we expected that the noise condition will be associated with higher beta-range SP and CMC than the condition without noise. This prediction would also be in line with studies showing that ON is characterized by higher local (within an area) and long-range (between areas) synchrony (Kitajo et al., 2003, 2004, 2007; Ward et al., 2006). The findings confirmed our predictions. The improved motor performance with ON was related to higher cortical motor SP and to higher CMC.

## Materials and methods

### Subjects

Eight healthy right-handed subjects (8 females, mean age 30.5 ± 14.35 years) without any history of neurological disease took part in the study. The handedness was assessed with the Oldfield questionnaire (Oldfield, 1971). All subjects had previously participated in similar experiments before. All subjects participated according to the declaration of Helsinki from 1964, with informed consent and approval of the local ethics committee.

### Experimental paradigm

#### Paradigm

**Figure 1:**
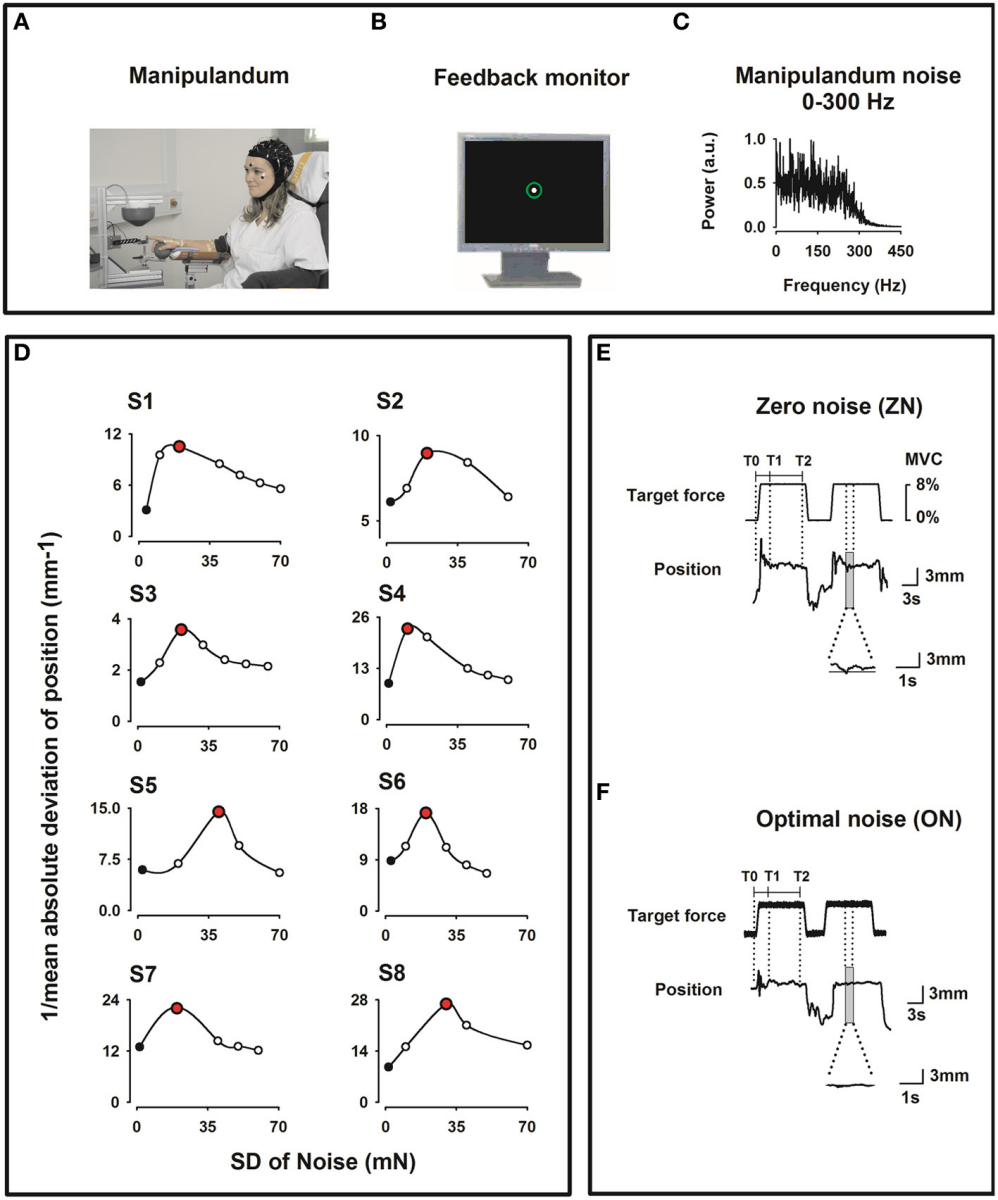
**Experimental setup. (A)** Home-made index finger manipulandum producing a target static force (8% of individual maximum voluntary contraction) on which noise in the frequency bandwidths 0–300 Hz is added. Profile of the target static force in E. EEG (41 channels), electrooculogram (EOG), and EMG from the right first dorsal interosseus (FDI), the right flexor digitorium superficialis (FDS) and the right extensor digitorum communis (EDC) muscles were recorded. **(B)** Visual feedback of the finger position as a solid white dot within a green circle indicating the tolerance for position errors, displayed on a monitor in front of the subject. **(C)** Spectral power of the noise of the manipulandum in arbitrary units (au) for the 0–300 Hz frequency bandwidth. **(D)** Effect of the SR on the motor performance for all subjects recorded prior to the experimental session and computed as the inverse of the mean absolute deviation (1/MAD) of the finger position. Note the inverted U-shape like curve. During the experimental session only two noise levels were individually chosen, i. e. zero noise (ZN, black filled dots) and optimal noise (ON, red filled dots). **(E,F)** Original curves for target force and finger position (representing the exerted force) for ZN **(E)** and ON **(F)** for the frequency bandwidth noise 0–300 Hz. Transitory phase of the task between markers T0 and T1 and stationary phase between markers T1 and T2. Note in the magnified position traces the better performance for ON than for ZN.

During the experiment, the subject sat in an electrically shielded, dimly lit room. The right arm was supported by a splint and the subject was instructed to place the right hand over a sphere and the index finger in the ring of a home-made manipulandum (see Figure 1A).

We employed the same manipulandum as in Trenado et al. (2014). The manipulandum was designed to produce a vertical force in the upward direction on the ring. The subject had to compensate this vertical force, called target force (Figure 1E) and maintain it by applying force pseudo-isometrically at the level of the metacarpophalangeal joint in the opposite direction (downwards).

#### Force profile

The target force shown in Figure 1E was set at 8% of the maximum voluntary contraction (MVC) which was determined for each subject prior to the experiment. We used low force as it has been shown that motor cortical neurons are most sensitive to forces within a low force range (Hepp-Reymond et al., 1989). Each trial comprised three phases: a 1 s *upward ramp phase* (rising cosine function to ensure a smooth start) followed by 12 s-period of SF, followed by 1 s *downward ramp phase* (decreasing cosine function) to ensure a smooth end of the generated force.

#### Visual feedback

The feedback of the force exerted by the subject was a white dot (radius 2 mm) within a fixed green circle displayed on a 19″ monitor placed 100 cm in front of him/her (Figure 1B). Thus, the feedback displayed was a positional one, since the finger position proportional to the force applied, was measured. The white dot moved within the fixed green circle (radius 6 mm including the line thickness of 2 mm) representing the range within which the white dot was allowed to move. When the force was applied to the ring and thus to the index finger, the subject had to compensate it by applying a force in the opposite direction and to maintain it pseudo-isometrically in the middle of the green circle. A finger displacement of 1 mm corresponded to 2.85 mm visual feedback. The tolerance for the positional errors was the green circle.

#### Experimental conditions

In the present experiment Gaussian noise in the range 0–300 Hz was continuously applied to the manipulandum and thus added to the target force. We choose this broad bandwidth because, in Trenado et al. (2014) this broad frequency bandwidth was associated with the best sensorimotor performance compared to a noise bandwidth 0–15 Hz and of 250–300 Hz and also was most pleasant.

Figure 1C shows the SP of the noise. The noise was generated by a MATLAB customized program which enabled various amplitude levels of noise intensity up to high noise (approx. 200 mN) (for more details see Trenado et al., 2014).

Prior to the experiment the following tests were performed:

*First*, the subjects performed a few trials to get familiarized with the task and learn “what” to do and “how” to do it.
*Second*, we defined for each subject the noise level which could be considered as ON. In particular, we made use of a MATLAB customized program that delivered a force at 8% of the MVC during 110 s and added noise levels in an incremental fashion. Immediately after that we calculated the performance as a function of the noise level, i.e., the SR curve. Figure 1D shows the performance inverted U-like curve, which is the signature of the SR, as function of noise intensity for all subjects. We defined the ON as the noise level inducing the best performance, as measured by the smallest absolute deviation from 0 (the highest value of the inverse of the mean absolute deviation, 1/*MAD*). The procedure was repeated five times to ensure reliability of the measures.

During the experimental session, 10 recording series of five trials each were collected for each experimental condition (zero noise, ZN and ON) thus reaching 50 trials for each of them. The two conditions, ZN and ON, were presented in a pseudo-randomized fashion.

To ensure that subjects sustained their attention during the experiment, they had to report after each series of five trials whether the trials were with or without added noise. The subjects were instructed to avoid any other movements and to fix their gaze on visual feedback during the trials.

To avoid fatigue, rest intervals of 5–7 s were included between trials. Rest periods of about 5 min between the series were given to avoid adaptation to the perception of the noise (Berglund and Berglund, 1970).

At the end of the experimental session the subjects reported whether the noise helped them to be more precise.

### Recordings

The EEG (bandpass DC-500 Hz, sampling rate 2000 Hz) was recorded (SynAmps 2, NeuroScan, El Paso, TX, USA) from 41 scalp positions referenced to Cz with ground at FzA, accordingly to the 10/10 system (Figure 1A). Electrode impedances were kept under 5 kOhm. The electrooculogram (EOG, same bandpass and sampling rate as for EEG) was recorded to exclude data segments contaminated with eye movements for further analysis. Electromyographic activity (EMG, bandpass DC-500 Hz; sampling rate 2000 Hz) was recorded with surface electrodes using a belly-tendon montage from the pars indicis of the right flexor digitorium superficialis (FDS), the right first dorsal interosseus (FDI), and the right extensor digitorum communis (EDC). Our task requires synergetic co-contraction of these three muscles, which have intermingled cortical representations (Maier and Hepp-Reymond, 1995a,b; Schieber, 2002; Spinks et al., 2008).

The force and displacement of the finger were recorded in parallel with the electrophysiological data (same bandpass and sampling rate as for EEG). Data were stored and analyzed off-line.

### Data analysis

#### EEG-EMG coherence analysis

For the analysis, markers were put on the force trace of both conditions (Figures 1E,F): T0 at the beginning of the ramp phase of the SF, T1 at 4 s after it and T2 at 8 s after T1. Only data corresponding to the stationary phase of the SF between T1 and T2, i.e., a period of 8 s duration, were taken into consideration in the analysis. This was done to avoid transient effects during the period T0-T1. For ZN and ON data from the same muscle were taken for the analysis.

The ZN and ON data from all trials were concatenated separately. Within each condition, data was further cut into non-overlapping segments of 512 ms length allowing a frequency resolution of 1.96 Hz. Artifact rejection was visually performed off-line trial-by-trial to exclude segments contaminated with eye movements. Based on previous observations large positional deviations occurred also as a result of unexpected muscle contraction and lack of attention. Segments, in which the white point exited the green circle, were excluded from further analysis. They represented approximately 1% of the total number of segments for each experimental condition.

The EEG signal was then transformed into the reference free current source density (CSD) distribution which approximates the underlying cortical activity (Nunez et al., 1997). The CSD algorithm was computed using the spherical spline interpolation method (Perrin et al., 1989) as implemented in the commercial software “Brain Vision 2.0.2” (München, Germany). EMG signals were first high-pass filtered at 30 Hz to avoid artifacts and subsequently rectified.

The discrete 512 points Fourier transform was computed for each segment of the whole 0–500 Hz frequency range. Hundred fifty artifact-free segments per condition were selected and analyzed for each subject to base the analysis on the same number of segments per condition as in previous studies. For all subjects, data were pooled separately for each condition, and CMC, SP, and performance were compared between conditions.

#### Calculation of EEG spectral power (SP) and EEG-EMG coherence (CMC)

The maximum EEG SP was found over C3 in six subjects and over C1 in two subjects. It was also located where the maximum value of the EEG-EMG coherence was obtained with the muscle which was most constantly activated during the task. In four subjects the maximum coherence was found with FDI, in two subjects with the EDC and in two subjects with FDS.

*SP* for a given channel (*c*) was calculated according to the following equation

(1) $$SP_{c}(f)=\frac{1}{n}\sum_{i=1}^{n}C_{i}(f)C_{i}^{*}(f),$$

where *C*_*i*_ represents the Fourier transformed channel *c* for a given segment number (*I* = 1,…, *n* = 150) and “^*^” denotes the complex conjugate.

Coherence values were calculated between the EEG channels corresponding to the primary sensorimotor area contralateral to the active finger and the rectified EMG. Coherence values were calculated on the basis of the following formulae:

(2) $$Coh_{c1,\;c2}(f)=\frac{{\left|S_{c1,\;c2}(f)\right|}^{2}}{\left|SP_{c1}(f)\right|\left|SP_{c2}(f)\right|},$$

where

(3) $$S_{c1,\;c2}(f)=\frac{1}{n}\sum_{i=1}^{n}C_{1i}(f)C_{2i}^{*}(f),$$

here *S*_*c*1, *c*2_(*f*) denotes the cross-spectrum for the EEG signal channel *c*1 and the rectified EMG signal in channel *c*2 at a given frequency *f* and *SP*_*c*1_(*f*) and *SP*_*c*2_(*f*) denote the respective SP for *c*1 and *c*2 at the same frequency.

For frequency *f*, the coherence value *Coh*_*c*1, *c*2_(*f*) corresponds to the squared magnitude of a complex correlation coefficient. The function *Coh*_*c*1, *c*2_(*f*) is a real number between 0 and 1.

Coherence between a pair of signals was considered to be significant if the resulting value lies above the confidence level (*CL*) (Rosenberg et al., 1989).

(4) $$CL(\alpha )=1-{\left(1-\alpha \right)}^{\frac{1}{n-1}},$$

where *n* is the number of segments and α is the desired level of confidence. Coherence values were significant when they lied above the 95% confidence limit. For 150 segments the 95% CL for each subject was 0.019. Following the approach introduced by Evans and Baker (2003) to determine whether the grand-average coherence is significantly different from zero, we firstly calculated the probability density function of a single-subject coherence values under the null hypothesis of no-coupling by using the formula:

(5) $$\mathrm{Pr}ob(Coh)\;=(1-N){\left(1-Coh\right)}^{N-2},$$

which is obtained by differentiating the formula for the cumulative probability of coherence given in Rosenberg et al. (1989). The probability density of the grand-average coherence was estimated by convolution of the individual coherence densities. The 95% percentile of this probability distribution function was used as a significant level for the averaged coherence (*P* < 0.05), which yields 0.023.

#### Calculation of performance

To test the effects of the SR phenomenon on the finger performance, the mean absolute deviation (MAD) was computed on the basis of the formula:

(6) $$MAD\;=\frac{1}{n}\sum_{i=1}^{n}\left|x_{i}\right|,$$

where *x*_*i*_ is the value of finger position relative to the applied force at the sampling point *i*. *MAD* measures the deviation amplitude of the white dot relative to the center of the green circle during the application of the force on the manipulandum in the y-direction (upwards and downwards).

### Statistical analysis

#### Statistical analysis of the EEG spectral power (SP)

To test for statistical difference in cortical SP between ZN and ON, we measured the individual areas under the SP curve, *A*_*pow*_. The frequency windows for EEG *A*_*pow*_ were 15–45 Hz (beta and gamma ranges), 15–30 Hz (beta-range), and 30–45 Hz (gamma-range). To prepare data for statistical analysis, data were logarithmically transformed to yield symmetric distributions according to the formula:

(7) $${A^{\prime }}_{Pow}=\log_{10}\left(0.001+A_{Pow}\right)-\log_{10}(0.001),$$

where the first *value* (0.001) has been selected to fulfill: (a) homogeneity of variance, and (b) symmetry of distribution. The second one [log_10_ (0.001)] was defined in such a way that the transformation maps 0 to 0.

#### Statistical analysis of CMC

To test for statistical difference in CMC between ZN and ON, we measured the individual area under the coherence curve and above the significance level, *A*_*coh*_. The considered frequency windows for the *A*_*coh*_ were 15–30, 15–45, and 30–45 Hz.

To prepare these data for the statistical analysis, individual values for *A_*coh*_* were first transformed logarithmically to yield symmetric distributions according to the formula:

(8) $${A^{\prime }}_{CMC}=\log_{10}\left(0.002+A_{CMC}\right)-\log_{10}(0.002),$$

where the first *value* (0.002) has been selected to fulfill: (a) homogeneity of variance, and (b) symmetry of distribution. The second one [log_10_(0.002)] was defined in such a way that the transformation maps 0 to 0.

#### Statistical analysis of performance

To account for the inter-subject variability and achieve symmetry on the data distribution, the *MAD* values were also first logarithmically transformed according to the formula:

(9) $$MAD^{\prime }=\log_{10}(100000+MAD)-\log_{10}(100000),$$

where the first *value* (100000) has been selected to fulfill: (a) homogeneity of variance, and (b) symmetry of distribution. The second one [log_10_ (100000)] was defined in such a way that the transformation maps 0 to 0.

For EEG SP, CMC, and performance paired Wilcoxon test was performed with the null hypotheses that the differences of the means between ZN and ON were zero.

To investigate changes in the performance, SP and CMC between both conditions Spearman correlation coefficient was computed.

## Results

### Behavioral performance

All eight subjects performed the task according to the instructions. None of them reported fatigue or anxiety during the experimental session. All subjects reported that they felt the ON during the experiment.

**Figure 2:**
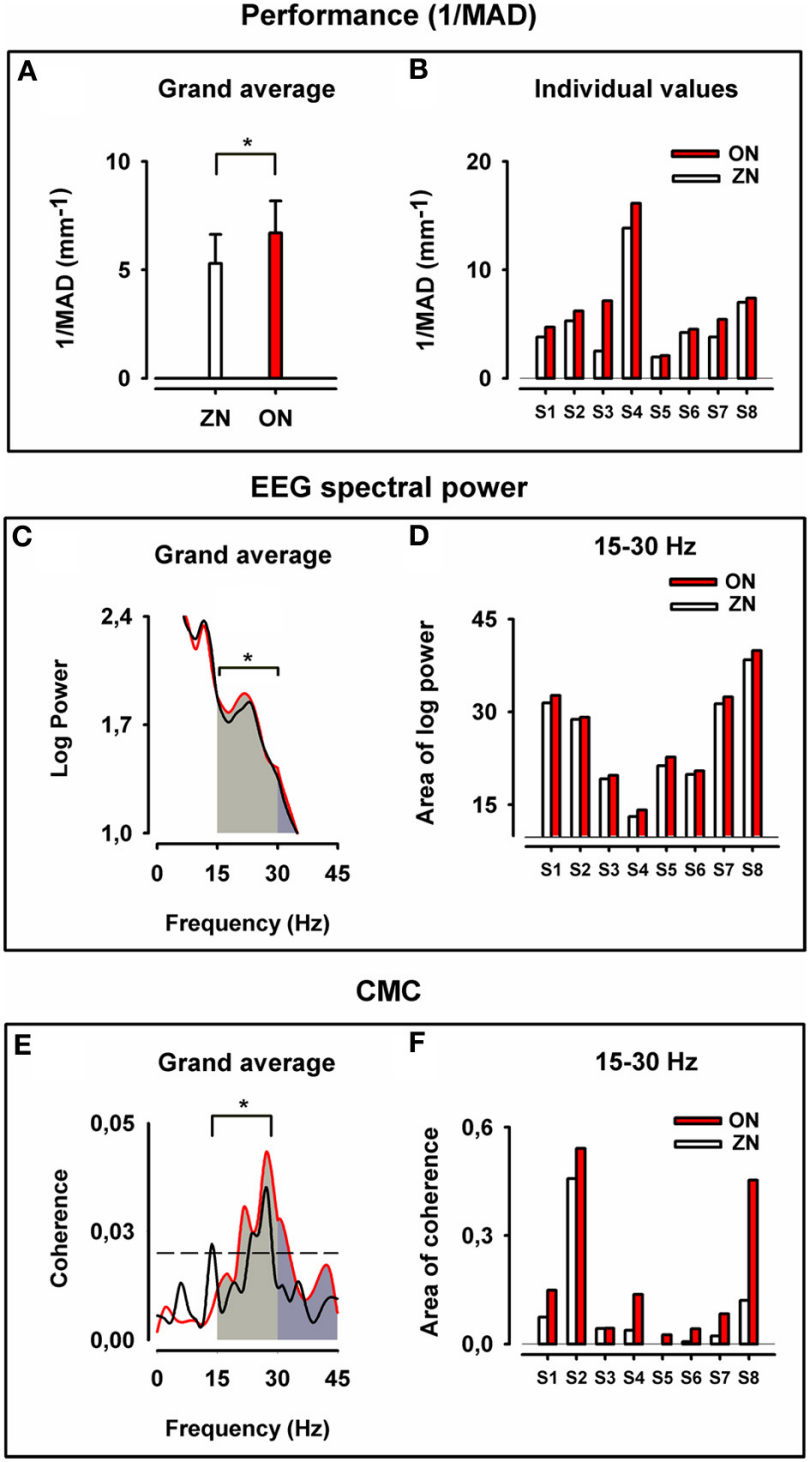
**Motor performance, cortical motor spectral power and corticomuscular coherence for zero noise (ZN, black) and optimum noise (ON, red)**. Upper panel: Grand average of the inverse of the mean absolute deviation (1/MAD) of the finger position in **(A)** and individual values of (1/MAD) in **(B)**. Note the stochastic resonance (SR) effect with better performance for ON (^*^p = 0.008). Middle panel: Grand average for cortical motor log SP in **(C)** for ZN and ON and plots of the individual values of area under the curve of log SP for both conditions in beta range **(D)**. Note the stronger cortical motor log power for beta-range (^*^p = 0.008). Lower panel: Grand average of CMC in **(E)** and individual plots for all values (areas under the curve and above the significance level) in beta range **(F)**. Note the higher CMC for ON in beta-range (^*^p = 0.008). In **(E,F)** for ZN and ON data from the same muscle were taken for the analysis.

The 1/MAD values were significantly higher (which means better performance) in the ON than in the ZN condition, as can be seen in the grand average of the performance displayed in Figure 2A and in the individual 1/MAD values for both conditions in Figure 2B (*p* = 0.008, Wilcoxon paired test; *N* = 8 throughout the whole text).

### EEG spectral power (SP) over the motor cortex

From the grand average of log SP curves (Figure 2C) and individual SP values (Figure 2D), the ON was characterized by significantly higher cortical motor SP (*p* = 0.008, Wilcoxon paired test) in beta-range. The difference was also significant in beta-gamma range (15–45 Hz) (*p* = 0.008, Wilcoxon paired test), but not for gamma-range only (30–45 Hz).

The high SP in the beta-range during ON indicates stronger cortical motor synchrony.

### Corticomuscular coherence (CMC)

All eight subjects exhibited CMC for both ZN and ON and six of them had CMC in the beta-range. For the other two subjects the maximum peak of CMC was at the border of the gamma-range. For all subjects the maximum CMC occurred over the left sensorimotor cortex, particularly at C3 (6/8) and at C1 (2/8). The highest CMCs occurred with the FDI (4/8), EDC (2/8), and FDS (2/8).

Figure 2 displays the grand average CMC curves (E) and the individual values of coherence for both conditions in beta-range (F). It is noticeable that ON had higher CMC than the ZN (*p* = 0.008, Wilcoxon paired test) for the beta-range. The difference between conditions for the gamma-range (30–45 Hz) is at the border of significance (*p* = 0.08).

The high CMC in the beta-range during ON indicates a stronger corticospinal synchronization during ON than during the ZN condition.

The statistical analysis gives support for the hypothesis that the improved performance during ON is consistent with to increased beta-range SP and beta-range CMC.

No significant correlations between improvement in performance and increase in SP and CMC in ON were found.

## Discussion

Here we show that SR generated by an optimal external tactile-proprioceptive stimulus applied on the fingertip improves performance in a visuomotor task. This improvement was consistent with an increase of cortical motor SP and of corticomuscular (EEG-EMG) coherence (CMC) in the beta-range. These findings provide evidence for local and long-range synchronization as neural correlates of SR in the sensorimotor system.

### Stochastic resonance is related to higher cortical motor and corticospinal synchrony

It is well known that synchronization subserves the selective and effective transmission of information in the neuronal networks involved in sensorimotor integration (Fell and Axmacher, 2011; Siegel et al., 2012). As suggested by Mendez-Balbuena et al. (2012), an explanation of the improved performance in our task during optimal noise is related to an enhanced neuronal synchronization at spinal, cortical, and corticospinal level.

During SF control as required in our task, the oscillatory activity of cortical motor areas and contralateral spinal motoneurones are synchronized in the beta-range (15–30 Hz), as reflected in CMC. Recently, we have demonstrated that the EEG-EMG coherence was related to the precision of performance in a visuomotor task (Kristeva et al., 2007; Mendez-Balbuena et al., 2012). The improved precision in the pseudo-isometric compensation of a SF correlated with higher beta-range cortical motor SP and higher beta-range CMC. This implies that a better performance is related to more effective corticomuscular synchronization. Corticomuscular synchrony is not only an efferent phenomenon as Baker et al. (2006) provided direct evidence that during sensorimotor processing sustained afferent discharge from muscle receptors was coherent with central oscillations. In addition, applying directed coherence analysis, Witham et al. (2011) have demonstrated that both descending and ascending pathways contributed to corticomuscular coherence CMC. Hence, one can postulate that the ON applied in our study enhanced the sensitivity of cutaneous receptors, muscle spindle afferents, and Golgi tendon organs and thus improved the sensorimotor integration at spinal, cortical and corticospinal level. This leads to a stronger local cortical motor synchrony and motor cortex drive to the muscles and to a better performance. Stronger beta-range cortical motor synchrony, as reflected in higher beta-range SP and higher beta-range CMC, has been also shown by others to be associated with better performance (Baker, 2007; Pogosyan et al., 2009).

An unexpected finding was the absence of correlation between the improvement in performance and the increase in cortical motor SP and CMC during ON: This surprising finding strongly suggest that the performance improvement also depends on other factors besides of the application of external noise, e.g., different internal noise for the various subjects. As shown by Aihara et al. (2010) the SR in visual perception is substantially influenced by the internal noise within the brain. In our study the absence of correlations is probably due to high interindividual variability in both internal noise and generation of the corticomuscular coherence.

Our findings are consistent with data reported by Kitajo et al. (2007) who demonstrated that both detection of weak visual signals to the right eye and phase synchronization of EEG signals from widely separated areas of the human brain were increased by addition of weak visual noise to the left eye. They found a close relationship between the resulting noise-induced changes in behavioral performance and the changes in phase synchronization between widely separated brain areas. In the visual system, noise-induced SR also engaged modulation of EEG SP in the alpha band in the left occipital region (Mori and Kai, 2002). And in the human auditory system, Ward et al. (2010) provided evidence that intra- and interregional EEG neuronal synchronization are facilitated by the addition of moderate amounts of random noise.

To conclude, our findings showing that in the sensorimotor system ON that maximizes performance is consistent with increase in cortical motor SP and in the corticomuscular coherence in beta-range, contribute to the understanding of the physiological mechanisms of the SR phenomenon.

## Author contributions

Carlos Trenado, Elias Manjarrez, Jürgen Schulte-Mönting, Ignacio Mendez-Balbuena, Frank Huethe, Marie-Claude Hepp-Reymond, and Rumyana Kristeva designed the experiment and the methods. Carlos Trenado, Rumyana Kristeva, Ignacio Mendez-Balbuena carried out the experiment. Carlos Trenado, Bernd Feige, Jürgen Schulte-Mönting, and Rumyana Kristeva analyzed experimental data. Rumyana Kristeva, Carlos Trenado, Elias Manjarrez, Ignacio Mendez-Balbuena, Jürgen Schulte-Mönting, Frank Huethe, Bernd Feige, Marie-Claude Hepp-Reymond interpreted data and wrote the manuscript. All authors approved the manuscript.

### Conflict of interest statement

The authors declare that the research was conducted in the absence of any commercial or financial relationships that could be construed as a potential conflict of interest.
